# Longitudinal Evaluation of PD-L1 Expression on Circulating Tumor Cells in Non-Small Cell Lung Cancer Patients Treated with Nivolumab

**DOI:** 10.3390/cancers13102290

**Published:** 2021-05-11

**Authors:** Mio Ikeda, Yasuhiro Koh, Shunsuke Teraoka, Koichi Sato, Jun Oyanagi, Atsushi Hayata, Nahomi Tokudome, Hiroaki Akamatsu, Yuichi Ozawa, Katsuya Endo, Masayuki Higuchi, Masanori Nakanishi, Hiroki Ueda, Nobuyuki Yamamoto

**Affiliations:** 1 Internal Medicine III, Wakayama Medical University, Wakayama 641-8509, Japan; ikdmo30@gmail.com (M.I.); teraoka@wakayama-med.ac.jp (S.T.); sato-k@wakayama-med.ac.jp (K.S.); joyanagi@wakayama-med.ac.jp (J.O.); atsushih@wakayama-med.ac.jp (A.H.); ntokudom@wakayama-med.ac.jp (N.T.); h-akamat@wakayama-med.ac.jp (H.A.); u1.ozawa@wakayama-med.ac.jp (Y.O.); masa-n@wakayama-med.ac.jp (M.N.); hu11@wakayama-med.ac.jp (H.U.); nbyamamo@wakayama-med.ac.jp (N.Y.); 2 Medical Business Sector, Hitachi Chemical Co., Ltd., Chikusei 308-0861, Japan; kat-endou@hitachi-chem.co.jp (K.E.); m-higuchi@hitachi-chem.co.jp (M.H.)

**Keywords:** liquid biopsy, circulating tumor cells, lung cancer, PD-L1, nivolumab

## Abstract

**Simple Summary:**

Programmed death-ligand 1 (PD-L1) expression in tumor tissue is a predictor for the efficacy of immune checkpoint inhibitors. We have previously reported that PD-L1 positive rate on circulating tumor cells (CTCs) in non-small cell lung cancer patients at baseline was correlated with response to nivolumab. Here, we sequentially evaluated PD-L1 expression on CTCs in 45 enrolled patients at baseline and week 4, 8, 12 and 24 or progressive disease (PD). The median of PD-L1-positive CTC number between baseline and week 8 were significantly different (*p* < 0.05), and progression-free survival was significantly longer in patients with ≥7.7% PD-L1 positivity rates (*n* = 8) than in those with <7.7% rates (*n* = 8; *p* < 0.01) at week 8. Our findings suggest that PD-L1 expression on CTCs during nivolumab treatment may be predictive of long-term efficacy.

**Abstract:**

Although programmed death-ligand 1 (PD-L1) expression on tumor tissue is a validated predictive biomarker for a PD-1 pathway blockade in non-small cell lung cancer (NSCLC), longitudinal changes in its expression during treatment remains elusive. Circulating tumor cells (CTCs) are assumed to reflect the transition of characteristics of the primary tumor undergoing anticancer treatment. Here, we sequentially evaluated the PD-L1 expression on CTCs in NSCLC patients treated with nivolumab. Forty-five patients were enrolled, and CTCs were enriched from 3 mL of peripheral blood using a microcavity array system at baseline and weeks 4, 8, 12, and 24 or until progressive disease. The effective responses to therapy were compared between patients without progressive disease (PD) at week 8 (i.e., non-PD patients) and in those with PD between weeks 4 and 8 (PD patients) in terms of increased vs. decreased or equal CTC status at week 8 (for non-PD patients) or at the point of PD (for PD patients) compared to the baseline. Significantly more non-PD patients were classified as decreased or equal in number and proportion to PD-L1-positive CTCs among the detected CTCs (PD-L1 positivity rates) (*p* < 0.05). Moreover, progression-free survival was significantly longer in patients with ≥7.7% PD-L1 positivity rates (*n* = 8) than in those with <7.7% rates (*n* = 8; *p* < 0.01) at week 8. These results suggest the predictive significance of the early evaluation of PD-L1 expression on CTCs for maintaining the benefits from nivolumab treatment.

## 1. Introduction

The 5-year survival rate of patients with metastatic lung cancer after diagnosis was estimated to be only approximately 5% between 2008 and 2014 [1]. In the current decade, molecular-targeted therapy has opened up the promising prospect for treating non-small cell lung cancer (NSCLC) and other solid tumors with effective clinical responses [2,3,4]. As one of these therapies, treatment with the programmed death 1 (PD-1)/PD-ligand 1 (PD-L1) pathway blockade for NSCLC patients dramatically improved tumor regression in approximately 20% of patients [5,6,7,8] and the 5-year survival in approximately 16% of patients [9]. It has been clinically evaluated that the expression of PD-L1 on tumor tissue is useful as a predictive biomarker fin response to the PD-1 pathway blockade; however, the incidences of patients who respond to the PD-1/PD-L1 blockade without PD-L1 expression in their tumor tissue indicate the insufficiency of this predictive technique [10,11,12]. Since the heterogeneity of PD-L1 expression within tumor tissue occurs intrinsically and the cancer may evolve with dynamic molecular changes, a single tissue biopsy at a particular point provides limited information for the diagnosis and selection of an appropriate therapy [13,14,15]. Therefore, the longitudinal evaluation of PD-L1 expression on tumor tissue through serial collection at multiple time points is needed; however, the repetitive acquisition of tumor specimens is difficult owing to a high invasiveness.

As one of the promising approaches with easily and minimal invasiveness, it has been assumed that the expression of PD-L1 on circulating tumor cells (CTCs) may complement the diagnosis using a tumor tissue biopsy. CTCs are assumed to be detached cells from the tumor tissue that circulate in the bloodstream; therefore, CTCs detected from the peripheral blood should reflect the characteristics of solid tumors [16,17,18,19]. It has been reported that the expression of PD-L1 on CTCs from patients can be evaluated and potentially used as a predictor for the efficacy of chemotherapy, radiotherapy, and PD-1 blockade therapy for several kinds of solid tumors, including NSCLC [20,21,22,23,24,25]. However, multiple studies have reported that there is no correlation between the expression of PD-L1 in tumor tissue and CTCs from NSCLC patients [26,27,28]. Although these results are thought to indicate heterogeneity in the primary tumor tissue and CTCs, the clinical utility of the expression of PD-L1 on CTCs as a predictive marker needs to be carefully evaluated. Several studies have sequentially evaluated the association of a response to the PD-1 blockade with the number of CTCs or PD-L1 expression on CTCs in NSCLC patients [21,28,29]; however, almost every study evaluated approximately 10–20 cases, and there were few cases in which the blood samples were both frequently and longitudinally evaluated.

We previously analyzed the number of CTCs and the expression of PD-L1 on CTCs in 38 advanced NSCLC patients treated with nivolumab at the baseline and observed a significant difference between the rate of PD-L1-positive CTCs in the detected CTCs from patients and the response to nivolumab treatment [30]. Moreover, we employed an automated microcavity array (MCA) system for CTC detection. This system enriched CTCs from whole peripheral blood based on the differences in size and deformability and detected CTCs in lung cancer patients with higher specificity compared to that by the CellSearch system approved by the United States Food and Drug Administration [31,32,33].

In this study, we longitudinally evaluated the change in CTC status from the same cohort consisting of 44 advanced NSCLC patients receiving nivolumab treatment using the MCA system. In particular, our study evaluated the CTC status every 4 weeks at the earlier evaluation points. The results of this study show the prognostic significance of the number and proportion of PD-L1-positive CTCs from advanced NSCLC patients in the earlier phase after the initiation of nivolumab treatment.

## 2. Results

### 2.1. Longitudinal Evaluation of CTCs Detected in Patient Samples

**Figure 1:**
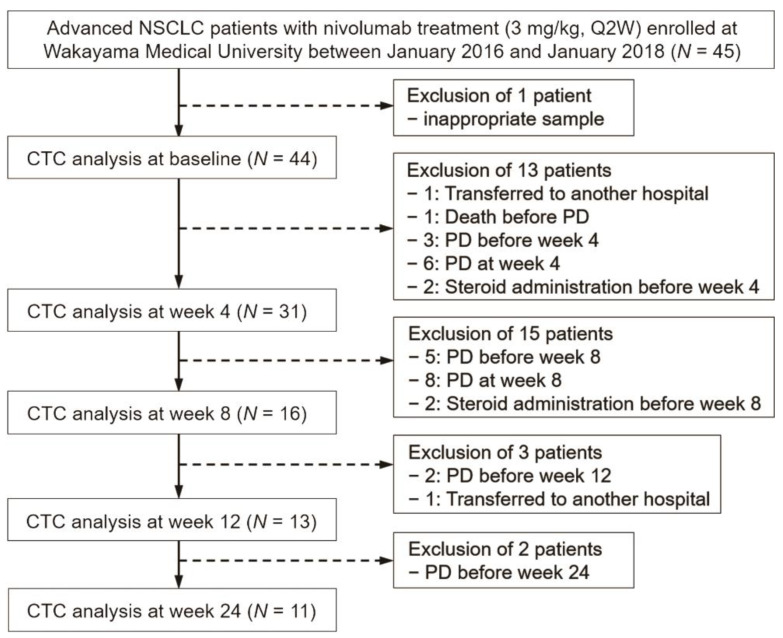
Flow diagram outlining the study.

In total, 45 advanced NSCLC patients were enrolled in this study at the Wakayama Medical University Hospital. One patient (N11) was excluded at the baseline because of an inappropriate sample. The detailed characteristics of the patients are shown in Table 1. Sequential analysis of the CTCs from the patients at the baseline and weeks 4, 8, 12, and 24 using the MCA system are shown in Figure 1.

**Figure 2:**
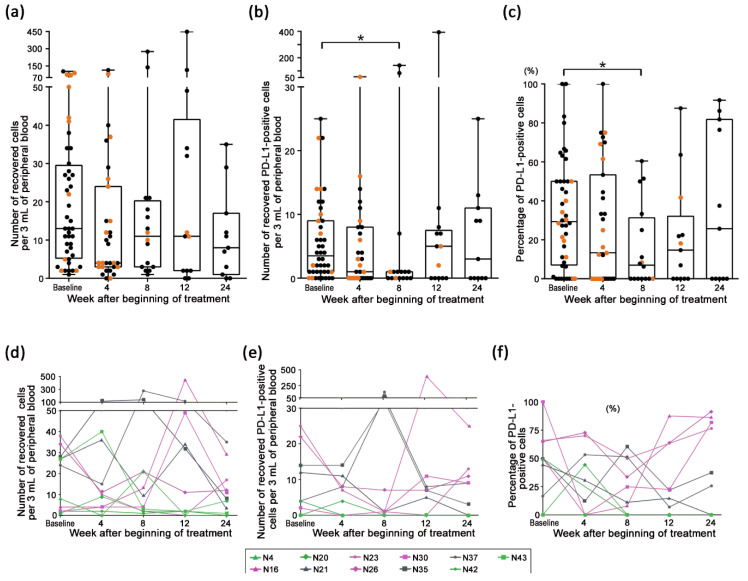
Longitudinal evaluation of the circulating tumor cells (CTCs) detected in the patient samples. (**a**) Distribution of the total number of CTCs from the patients at the baseline (*n* = 44), week 4 (*n* = 31), week 8 (*n* = 16), week 12 (*n* = 13), and week 24 (*n* = 11). (**b**) Distribution of the number of programmed death-ligand 1 (PD-L1)-positive CTCs from the patients at each evaluation point. * *p* < 0.05. (**c**) Distribution of the PD-L1 positivity rates in the patients at each evaluation point. * *p* < 0.05. Orange dots indicate the patients who were diagnosed PD during the next blood draw. Transition of the number of CTCs (**d**), number of PD-L1-positive CTCs (**e**), and PD-L1 positivity rates in the detected CTCs (**f**) from the patients receiving nivolumab treatment for more than 24 weeks (*n* = 11). The patterns of the CTC status change were classified into three types. Green indicates the first type (N4, N20, N42, and N43); pink indicates the second type (N16, N23, N26, and N30); and gray indicates other type (N21, N35, and N37).

Figure 2a,b shows the longitudinal transition of the total number of circulating CTCs and PD-L1-positive CTCs from each patient, respectively. Figure 2c shows the longitudinal transition of the PD-L1 positivity rates in the CTCs, which was defined as the percentage of the number of PD-L1-expressing CTCs to the total number of CTCs from each patient. Among those results, the data from 38 patients (N1–N39, except for N11) at the baseline overlapped with the previously reported data [30]. At the baseline, the CTCs were evaluated in 44 patients (median of number of CTCs, 13; range, 1–104), and PD-L1-positive CTCs were detected in 36 patients (82%) (median of percentage of PD-L1-positive CTCs, 29%; range, 0–100%); at week 4, evaluated in 31 patients (median of number of CTCs, 4; range, 0–115), and PD-L1-positive CTCs were detected in 18 patients (58%) (median of percentage of PD-L1-positive CTCs, 13%; range, 0–100%); at week 8, evaluated in 16 patients (median of number of CTCs, 11; range, 1–276), and PD-L1-positive CTCs were detected in 9 patients (56%) (median of percentage of PD-L1-positive CTCs, 7%; range, 0–60%); at week 12, evaluated in 13 patients (median of number of CTCs, 11; range, 0–449) and detected in 8 patients (62%) (median of percentage of PD-L1-positive CTCs, 15%; range, 0–88%); and at week 24, evaluated in 11 patients (median of number of CTCs, 8; range, 0–35) and detected in 6 patients (55%) (median of percentage of PD-L1-positive CTCs, 26%; range, 0–92%). The details of each patient are shown in Table S1. The median of the total number of CTCs at week 4 after the beginning of treatment tended to decrease compared to that at the baseline (*p* = 0.060), and the median of number of PD-L1-positive CTCs between the baseline and week 8 were significantly different (*p* < 0.05; Figure 2a,b). Moreover, a significant decrease was observed in the transition of PD-L1 positivity rates between the baseline and week 8 (*p* < 0.05) analyzed by the Wilcoxon matched-pairs signed rank test (Figure 2c). Figure 2d–f shows the change in CTC status at each time point, focused on the patients with continued nivolumab treatment for more than 24 weeks (*n* = 11). Although the results seem as though that there was no common feature in the transition of the number of CTCs and PD-L1 positivity rates in the CTCs, the trend of change in the number of PD-L1-positive CTCs and PD-L1 positivity rates could be classified into three types: First, the PD-L1 status was maintained at 0 cells and 0% at most evaluation points, and patients N4, 20, 42, and 43 belonged to this type; second, the PD-L1 status decreased once in the first half of the evaluation and increased in the latter half, and patients N16, 23, 26, and 30 belonged to this type; third, the transition of the PD-L1 status did not fit into either of those types. As another observation among the patients receiving nivolumab treatment for more than 24 weeks, the CTCs were still detected at week 24 in 82% of the patients (9 out of 11 patients), and 36% of patients (4 out of 11 patients) exhibited an increase in the total number of CTCs or PD-L1 positivity rates in the CTCs at week 24 compared to those at the baseline.

### 2.2. Correlation between the Change in CTC Status and Disease Progression

**Figure 3:**
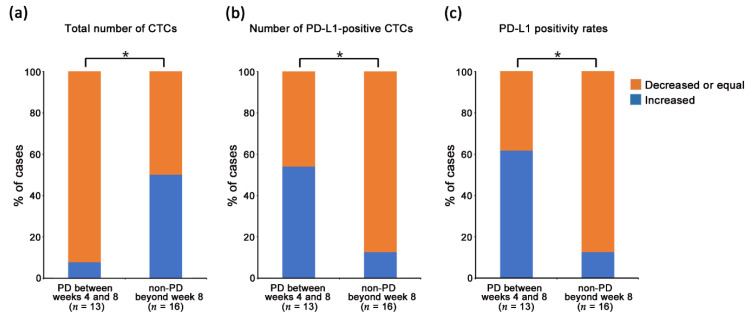
Comparison of the changes in the circulating tumor cell (CTCs) status between the patients who developed progressive disease (PD) between weeks 4 and 8 (*n* = 13) and who continued to benefit from nivolumab treatment (non-PD) beyond week 8 (*n* = 16). The increased vs. decreased or equal of each CTC status in PD patients was compared between the baseline and at the point of disease progression and, in non-PD patients, was compared between the baseline and at week 8. The association of changes in (**a**) the total number of CTCs, (**b**) number of programmed death-ligand 1 (PD-L1)-positive CTCs, and (**c**) PD-L1 positivity rates upon nivolumab treatment were compared between the PD and non-PD patients. Orange indicates that each CTC status at the point of disease progression or week 8 was decreased or equal compared to that at the baseline, while blue indicates an increase. * *p* < 0.05.

To validate the correlation between the CTC status and early disease progression, the total number of CTCs, number of PD-L1-positive CTCs, and PD-L1 positivity rates at the point of disease progression in PD patients until week 4 or 8 and at each evaluation point in the patients with an effective response to therapy (non-PD) beyond week 4 or 8 were compared with those at the baseline. The changes from the baseline were classified as increased, decreased, or equal. The PD patients included those diagnosed as PD before or at the timing of the scheduled blood draw after the last blood draw. The other patients were defined as non-PD. The proportions of the CTC status change classified as increased, decreased, or equal were compared between the PD and non-PD patients in Figure 3. Thirteen patients developed PD between weeks 4 and 8. Comparing the change in the proportion of the CTC status between patients who developed PD between weeks 4 and 8 and non-PD patients beyond week 8 (*n* = 16), the number of PD-L1-positive CTCs and PD-L1 positivity rates at week 8 compared to those at the baseline were significantly more “decreased or equal” in non-PD patients beyond week 8 than those at the point of disease progression in PD patients between weeks 4 and 8 (*p* < 0.05; Figure 3). However, the total number of CTCs at the point of disease progression in PD patients, compared to those at the baseline, were significantly more “decreased” than those in non-PD patients beyond week 8. On the other hand, seven patients developed PD with the data of the CTC status at the point of disease progression until week 4, and there were no significant differences in the changes in both the CTC counts and PD-L1 positivity rates between the PD and non-PD patients beyond week 4 (*n* = 31) (Figure S1). Moreover, only two patients developed PD between weeks 8 and 12, and the number of PD-L1-positive CTCs and PD-L1 positivity rates in the CTCs at the point of PD increased in one patient and decreased in another, compared to those at the baseline.

### 2.3. Correlation between PD-L1 Positivity Rates with the Efficacy of the Nivolumab Treatment

**Figure 4:**
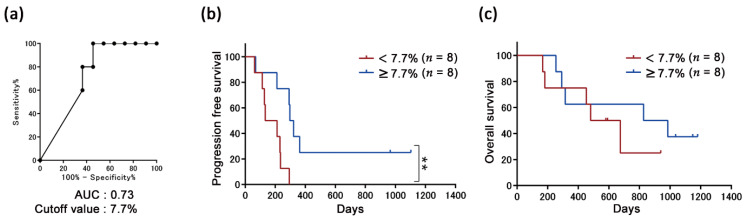
Prediction of the long-term efficacy based on the ratio of programmed death-ligand 1 (PD-L1)-positive circulating tumor cells (CTCs) at week 8. (**a**) Cutoff value of the PD-L1 positivity rates in the CTCs at week 8 to segregate the durable clinical benefits (DCB; *n* = 11) from the non-DCB (*n* = 5) calculated by the receiver operating characteristic (ROC) curve. (**b**) Kaplan–Meier curve for the progression-free survival (PFS). The PFS was significantly longer in patients with a ≥7.7% PD-L1 positivity rate (*n* = 8; median, 309.5 days) than that in patients with a < 7.7% PD-L1 positivity rate (*n* = 8; median, 172.5 days); ** *p* < 0.01. (**c**) Kaplan–Meier curve for the overall survival (OS). No significant difference in the OS between patients with ≥7.7% PD-L1 positivity rates (*n* = 8; median, 578 days) and in those with <7.7% PD-L1 positivity rates (*n* = 8; median, 906 days); *p* = 0.43.

We have previously reported, for the same cohort consisting of 38 patients, that progression-free survival (PFS) is significantly correlated with the PD-L1 positivity rates in the CTCs of patients at the baseline [30]. To validate the correlation between the PD-L1 positivity rates on the treatment and efficacy, the cutoff value of the PD-L1 positivity rates in the CTCs was calculated at week 4, 8, or 12 based on the receiver operating characteristic (ROC) curve to segregate the durable clinical benefits (DCB) from the non-DCB. We observed that PFS was significantly longer in patients with ≥7.7% PD-L1 positivity rate (*n* = 8) than that in patients with <7.7% PD-L1 positivity rate (*n* = 8) at week 8 (*p* < 0.01), whereas there was no significant difference in the overall survival (OS) (*p* = 0.43; Figure 4). However, there was no significant correlation between the PD-L1 positivity rates and response to nivolumab at weeks 4 and 12 (Figure S2).

### 2.4. Significance of the Transient Increase in the Number of CTCs from Patients Defined as PR

**Figure 5:**
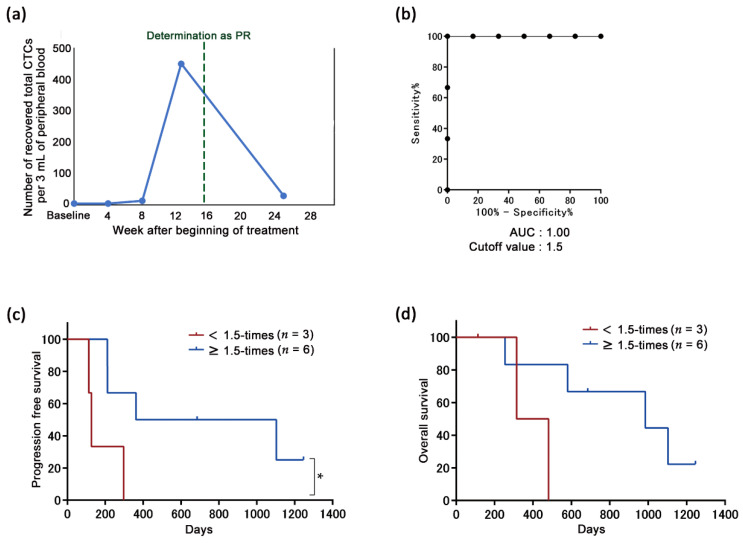
The correlation between the magnitude of the increase in the transient circulating tumor cells (CTC) prior to achieving a partial response (PR) and the duration of the response to the nivolumab treatment. (**a**) Representative example of the transition of the CTC counts and programmed death-ligand 1 (PD-L1) positivity rates in the patient sample (N16) achieving a PR. Blue line indicates the total number of CTCs. (**b**) Cutoff value of the magnification of the transient CTC increment prior to achieving a PR at the baseline to segregate the durable clinical benefits (DCB; *n* = 6) from the non-DCB (*n* = 3) according to the receiver operating characteristic (ROC) curve. (**c**) Progression-free survival (PFS) was significantly longer in patients with ≥1.5 times the increase in the CTC count (*n* = 6; median, 732.5 days) than in those with <1.5 times the increase in the CTC count (*n* = 3; median, 127 days); * *p* < 0.05. (**d**) Patients with ≥1.5 times the increase in the CTC count (*n* = 6; median, 985 days) had longer overall survivals (OS) than those with <1.5 times the CTC count (*n* = 3; median, 398 days); *p* = 0.075.

Interestingly, it was observed that the number of CTCs in seven out of nine patients, who were defined as PR, transiently increased immediately prior to achieving PR (Figure 5a). The cutoff value of the increased rates of the CTC counts, which was calculated by dividing the number of total CTCs prior to definition as PR by that at the baseline to segregate the DCB from non-DCB, was calculated to be 1.5 times according to the ROC curve (Figure 5b). The PFS was significantly longer in patients with ≥1.5 times the CTC count (*n* = 6) than in those with <1.5 times the CTC count (*n* = 3; *p* < 0.05; Figure 5c), and the OS showed the same tendency (*p* = 0.075; Figure 5d).

### 2.5. Transition of CTC Status in Patients Receiving Continued Nivolumab Treatment for More Than 24 Weeks

**Figure 6:**
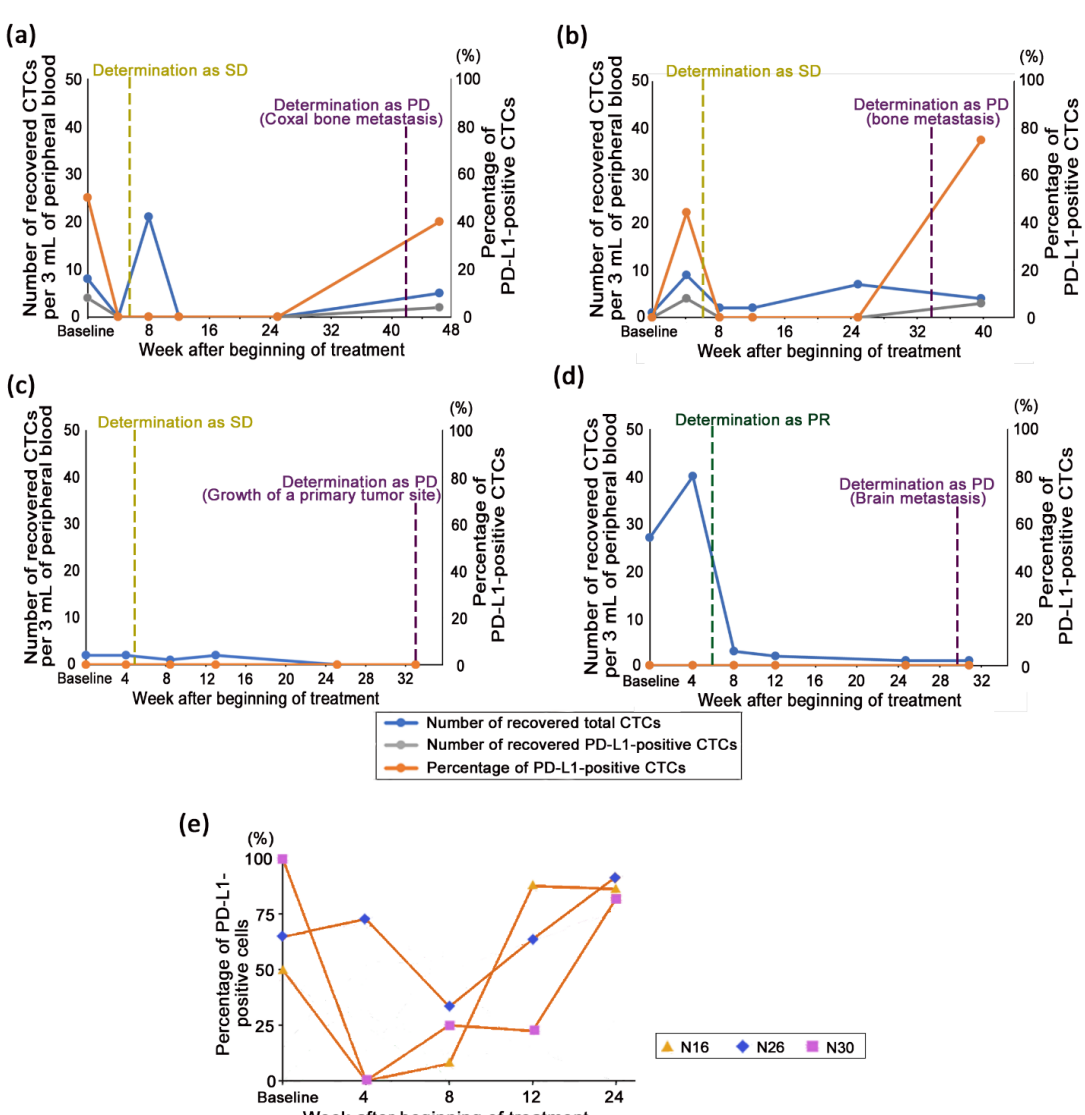
Longitudinal monitoring of the circulating tumor cell (CTC) status in patients benefiting from the treatment with nivolumab for more than 24 weeks. Representative examples of the transition of the CTC counts and programmed death-ligand 1 (PD-L1) positivity rates in patients during the nivolumab treatment from initiation. Blue line indicates the total number of CTCs, gray line indicates the number of PD-L1-positive CTCs, and orange line indicates the PD-L1 positivity rates in the CTCs. The transition of the CTC status in the patient samples, best response to treatment, progression-free survival (PFS), and overall survival (OS) were N4, stable disease (SD), 293 days, and 675 days, respectively (**a**); N20, SD, 235 days, and 939 days, respectively (**b**); N42, SD, 231 days, and 591 days, respectively (**c**); and N43, partial response (PR), 212 days, and 580 days (**d**), respectively. The transition of the PD-L1 positivity rates (**e**) in the patients who benefited from the nivolumab treatment for 12 months or more (N16, N26, and N30).

Among the 11 patients who continued nivolumab treatment for more than 24 weeks, nine patients were already defined as PD or deceased. Since one patient was deceased without definition as PD and four patients missed the blood draw at PD, four patients were subjected to a CTC analysis at the point of PD. Two patients were still non-PD until recently. Figure 6 shows the examples of the longitudinal change in the number of CTCs and PD-L1 positivity rates in four PD patients. All CTC changes were not correlated to the evaluation of the tumor tissue by CT imaging. Furthermore, the trends in the transitions of the CTC counts and PD-L1 positivity rates throughout the course of the treatment and at the point of disease progression compared to that at week 24 were different among the four patients. In patient N4, the tumor was determined as SD at day 38 without any change in the size of the primary tumor tissue compared to that at the baseline and remained stable until the appearance of a novel coxal bone metastasis at day 293, and CTCs were not detected from week 4 to 24, except for a transient increase in the CTC count at week 8. The nivolumab treatment was continued until day 324, and both the number of CTCs and PD-L1 positivity rates increased at this point compared to that at week 24 (Figure 6a). In patient N20, although the tumor was determined as SD at day 43 without any change in the size of the primary tumor tissue compared to that at the baseline and remained stable until the appearance of a novel lymph node metastasis and bone metastasis at day 235, the CTC counts increased or decreased at each evaluation point until week 24. However, PD-L1-positive CTCs were not detected, except for a transient detection at week 4. Although the last evaluation of CTCs was performed at day 278, the number of CTCs decreased compared to that at week 24, whereas the PD-L1 positivity rates increased drastically (Figure 6b). In patient N42, the primary tumor had 14% shrinkage at day 32, and the tumor gradually shrank until week 24. Throughout the course of the nivolumab treatment, there were few CTCs detected, and the PD-L1-positive CTCs were never detected. While disease progression was determined at day 231 due to growth of the primary tumor site, CTCs were not detected at the evaluation point of PD (Figure 6c). In patient N43, who underwent surgery of the primary tumor site, although CT imaging determined it as PR for lymph node metastasis at day 42 and remained stable until the appearance of a novel brain metastasis at day 212, the number of CTCs increased once at week 4, probably due to a temporary increase prior to achieving PR, and decreased continuously until week 24, following which, the number of CTCs during disease progression remained the same at week 24. Moreover, PD-L1-positive CTCs were never detected throughout the course of the treatment (Figure 6d). Interestingly, the PD-L1 positivity rates in patients N16, 26, and 30, who benefited from the nivolumab treatment for 12 months or more, were high at week 24 (range, 82–92%) (Figure 6e), while those at week 24 were kept low in four PD patients.

### 2.6. Transition of CTC Counts in Samples of Patients with Interrupted Treatment due to Immune-Related Adverse Events

It has been previously reported that there is some response to the PD-1 blockade with long-term clinical efficacy even after discontinuation of the treatment [9]. We evaluated the total number of CTCs from the patients who interrupted nivolumab treatment due to immune-related adverse events (irAE). A total of four patients had to interrupt the nivolumab treatment before week 8, and three of them maintained clinical efficacy for more than 6 months (N7, N18, and N29). Among them, patients N7 and N18 were still non-PD, and sufficient follow-up revealed a change in the CTC counts after discontinuation of the treatment. Although both patients underwent a transient increase of CTC counts prior to achieving PR, subsequently, the number of detected CTCs decreased (Figure S3). This needs be addressed in a larger cohort.

## 3. Discussion

In this study, we longitudinally evaluated the change in the number of CTCs and PD-L1 positivity rates in CTCs from 44 advanced NSCLC patients who received nivolumab treatment and analyzed the correlation between clinical findings and CTC status. To our knowledge, this is the first study to frequently evaluate CTC status in NSCLC patients receiving nivolumab treatment, particularly at early evaluation points. We previously reported that PD-L1 positivity rate in CTCs prior to nivolumab treatment is correlated with clinical efficacy, and the current study suggested the predictive significance of early evaluation of PD-L1 expression on CTCs during nivolumab treatment. The PD-L1 positivity rate in CTCs at week 8 after initiation of nivolumab treatment or immediate increase in the number of CTCs prior to achieving PR and the decrease thereafter were significantly correlated with response to nivolumab, proving the usefulness of over-time monitoring during treatment for diagnosis and prognosis. Although frequent tumor tissue biopsy is not appropriate, sequential evaluation of CTCs is a promising alternative approach with minimal invasiveness. Moreover, CTCs can be separated individually; thus, CTCs can be used for mutation and expression analyses of targeted protein to reflect intrapatient heterogeneity of the tumor [17].

In our study, there were three interesting observations at week 8 after beginning of the treatment. The first observation was that the ratio of PD-L1-positive CTCs decreased significantly compared to that at baseline (Figure 2C). The second was that the change in the number and ratio of PD-L1-positive CTCs at week 8, compared to those at baseline, were classified as decreased and equal in almost all non-PD patients beyond week 8 (88%), whereas the total CTC counts decreased in almost all PD patients between weeks 4 and 8 (92%) (Figure 3). Nicolazzo et al. reported that CTCs are detected in all NSCLC patients evaluated at 6 months after initiation of PD-1 blockade treatment, and patients harboring PD-L1-positive CTCs present a worse prognosis than those with PD-L1-negative CTC. However, such results could not be observed at 3 months after initiation of the treatment due to the very high proportion of PD-L1-positive CTCs among the detected CTCs and the lack of patients with PD-L1-negative CTCs [21]. Therefore, they discussed the possibility that PD-L1 expression on CTCs may have a clear predictive significance for clinical benefit later in the course of treatment, and the presence of PD-L1-positive CTCs at 6 months may reflect a mechanism of treatment escape. Several other studies have reported that the number of PD-L1-positive CTCs from patients with NSCLC or different gastrointestinal tumors determined as PD increase significantly compared to that at baseline within 2–3 months after beginning of PD-1 blockade therapy [28,34]. Our results seem to be consistent with these findings, and significant differences in changes in the status of PD-L1-positive CTCs were observed at week 8, suggesting the predictive significance of PD-L1 expression on CTCs at an earlier evaluation point after nivolumab treatment.

Moreover, our study also revealed that PFS was significantly longer in patients with ≥7.7% PD-L1 positivity rate (*n* = 8) than in those with <7.7% PD-L1 positivity rate (*n* = 8; *p* < 0.01) at week 8 as the third observation (Figure 4). This result seemed inconsistent with the first and second observations. However, among patients with <7.7% PD-L1 positivity rate, the rates of seven of eight patients were 0%; thus, it was speculated that tumor without PD-L1 upregulation responding poorly to nivolumab may increase in these patients, whereas PD-L1-expressing cancer cells disappeared until week 8. Although it may be important that the PD-L1 positivity rates at early phase of treatment become lower than those at baseline, it may be preferable to have a persistent number of cancer cells that respond to treatment for long-term efficacy.

Another observation for PD-L1 expression was noted in this study. PD-L1 positivity rates of all three patients who continued to benefit from nivolumab treatment for ≥12 months were high (range, 82–92%) at week 24. In addition, a trend was observed in the overall patient population that PD-L1 positivity rates once diminished until week 8 and then increased gradually thereafter (Figure 2C). These results suggest that elevation in PD-L1 positivity rates at the later phase of treatment was not a predictor of resistance but rather long-term efficacy of PD-1 blockade as opposed to that noted in the early phase of treatment. Dhar et al. reported that the proportion of PD-L1-positive CTCs from one patient with NSCLC who received avelumab treatment and achieved long-term efficacy for at least more than 600 days was always high (between 80.6% and 100%) during serial evaluation every few months until approximately day 350 [29]. However, our hypothesis conflicts with the previous report by Nicolazzo et al. [21]. As all these studies have a very limited sample size, further investigation is warranted.

As another prediction of long-term efficacy, immediate increase in CTC count prior to definition as PR also significantly correlated with long-term efficacy and survival (Figure 5). As a hypothesis of this increment, tumor tissue is drastically disrupted by the treatment, demonstrating its efficacy, and then a part of the collapsed tumor cells may invade into blood vessels. It is known that tumor cells are disseminated into blood during surgery [35,36]. In addition, similar phenomena have been reported using a mouse CTC model that the number of detected CTCs increase significantly within 10 days after chemotherapy or targeted therapy and decrease thereafter, and a part of the detected CTCs were morphologically determined to be living cells [37,38]. Among the six patients with a ≥ 1.5-fold transient increase in CTC counts prior to achieving PR, 3 patients were already determined as PD. Two of these 3 patients (N30 and 43) were determined to have disease progression due to distant metastasis, which may be caused by CTCs released prior to PR determination. By contrast, there was no trend between disease progression and the change in CTC count after long-term efficacy among patients (Figure 6), whereas it has been reported that increase in CTC counts is correlated with poor prognosis for patients undergoing chemotherapy in whom CTC counts are monitored longitudinally [39,40].

There are still a limited number of studies about the usefulness of PD-L1 expression on CTCs for diagnosis. Moreover, the method for CTC detection from peripheral blood is still developing, and there are differences in the detection rate and population of isolated CTCs depending on the methodology [41]. It has been noted that even the CellSearch system, established as CTC detection method for some kinds of cancer, may miss CTCs undergoing epithelial-to-mesenchymal transition (EMT) because of the dependency on expression of the epithelial marker EpCAM. Moreover, antibodies for detection of PD-L1 expression are not unified; therefore, differences in the staining properties are also considered. As discussed above, our findings in the current study also cannot escape such limitation. In addition, our analysis was performed on in a small patient cohort from a single institution. Therefore, we cannot eliminate the possibility that the significance of our study and some numerical number, such as cutoff values from ROC curve, may only fit to the current population. To demonstrate the significance of the change in PD-L1 expression on CTCs as predictive prognostic factor, our hypothesis should be further validated in another independent cohort with a larger sample size. Besides, there are a few limitations in this study besides the small sample size. First, criterion for CTC enumeration of this study employed cytokeratin (CK); therefore, CTCs without CK expression would not be counted due to the significant reduction in expression, although an automated MCA system is more suitable for CTC detection from patient with NSCLC compared to that with the CellSearch system [33]. Indeed, few nuclei-positive, CK-negative, CD45-negative, and PD-L1-expressing cells were observed in some cases. It has been suggested that presence of CTC undergoing EMT is predictably a poor prognosis for patients with solid tumor [42,43,44,45]. Moreover, EMT is thought to be associated with resistance to many kinds of cancer therapy, including immunotherapy and targeted therapies [46,47]. There was no trend of predictive significance of disease progression for patients with long-term efficacy (Figure 6), and it may indicate that cells undergoing EMT were missed. Second, as the detected number of CTCs from most patients were very low, it can be difficult to evaluate the exact PD-L1 positivity rate. There were some evaluation points at which only one or two CTCs were detected, and in such cases, PD-L1 positivity rate had to be mandatorily calculated as 0% or 100% in case of one CTC and 0%, 50%, or 100% in the case of two CTCs. It has been previously validated that an effective and simpler way for determining incremental increases in CTC detection from whole blood is by increasing the sample volume [48]. The blood volume of 3 mL for each analysis may not be enough for recent analysis. Third, it remains unclear whether the change in the status of PD-L1 expression on CTC correlates to changes in the tumor tissue. Although repetitive acquisition of specimen is difficult, paired sample collection of tissue at baseline and PD is necessary to validate this issue.

Our study indicated the importance of sequential evaluation of CTCs from advanced NSCLC patients. It has been suggested that analysis of PD-L1 expression on CTCs, particularly in the early phase of nivolumab treatment, may be useful for prediction of treatment efficacy. These results supporting the concept of the usefulness of evaluation of PD-L1 expression on CTC warrant further investigation.

## 4. Materials and Methods

### 4.1. Study Design

All subjects gave their informed consent for inclusion before they participated in the study. The study was conducted in accordance with the Declaration of Helsinki, and the protocol was approved by the Institutional Review Board at Wakayama Medical University (#1513, 14 November 2014). Forty-five advanced NSCLC patients receiving nivolumab monotherapy (3 mg/kg, Q2W) were enrolled in this study between January 2016 and January 2018 at Wakayama Medical University. For CTC detection, 3 mL of peripheral blood was collected in blood collection tubes (Becton Dickinson and Company, Franklin Lakes, NJ, USA) containing EDTA to prevent coagulation at baseline and week 4, 8, 12, and 24 or until PD from patients receiving nivolumab treatment. Blood samples were processed by the MCA system within 3 h after blood draw. Tumor responses were classified into CR, PR, SD, or PD based on RECIST v1.1. Patients who were not yet defined as PD at each evaluation points were classified as non-PD for that point. In addition, efficacy was also evaluated by DCB, indicating patients who maintained PR and SD for more than 6 months, or non-DCB. Patients that met any of the following criteria were excluded from serial evaluation: patients who received administration of systemic corticosteroids, patients who died during treatment, patients who were transferred to other hospitals, and patients who were defined as disease progression. OS and PFS were evaluated from the beginning of nivolumab treatment until last follow-up or death of the patient or disease progression. The last follow-up date of this study was at the end of June 2019. This study was approved by the institutional review board at Wakayama Medical University, and written informed consent was obtained from all donors. This study has been registered with the University Medical Hospital Information Network (UMIN) Clinical Trials Registry under the identifier UMIN000024414.

### 4.2. Tumor Cell Enrichment and Detection

CTCs were enriched and immunostained using an automated MCA system [30,31,32]. Peripheral whole blood sample was added to the reservoir of the MCA system, and then the blood sample was filtered through the metal filter in the cartridge. After tumor cells were captured, the cells were automatically stained for CD45, PD-L1, CK, and 4′,6-diamidino-2-phenylindole (DAPI). The reagents for CD45, CK, and DAPI staining were provided by Hitachi Chemical Company (Chikusei, Japan). PD-L1 was stained with 1:1000 diluted anti-PD-L1 rabbit mAb (28–8) (ab205921; Abcam, Cambridge, MA, USA) and then incubated with 1:500 diluted goat-anti-rabbit Alexa Fluor 647 (A-21245; Thermo Fisher Scientific, Waltham, MA, USA). An image of the entire cell array area was captured using a fluorescence microscope (Axio Imager M2m; Carl Zeiss, Oberkochen, Germany) with an integrated 10× objective lens and a computer-operated motorized stage, a digital camera (AxioCam 503 mono; Carl Zeiss), and ZEN image acquisition software (Carl Zeiss). CTCs were defined as DAPI, CK-positive, and CD45-negative cells. PD-L1-positive or -negative CTCs were counted by two people independently for each acquired image. The proportion of PD-L1-positive CTCs in the detected total CTCs were defined as PD-L1 positivity rate for each sample.

### 4.3. Statistical Analysis

Statistical analyses were performed using GraphPad Prism 6 (GraphPad Software, San Diego, CA, USA) and Wilcoxon matched-pairs signed rank, Mann–Whitney U, Fisher’s exact, and log-rank tests were performed accordingly. For the analyses of CTC count, cases harboring no CTC were classified as 0 cells. For the analyses of PD-L1 positivity rates, cases harboring no PD-L1 positive CTC was detected were evaluated as 0%, whereas cases harboring no CTC were excluded from the evaluation of PD-L1 positivity rates. *p*-value less than 0.05 was considered statistically significant.

## 5. Conclusions

Our study indicated that evaluation of longitudinal changes in the CTC status from prior to treatment to disease progression is informative for the assessment of clinically relevant information. The prognosis of patients treated with nivolumab may be predictable by evaluating the PD-L1 expression on CTCs at week 8 and comparing with that at the baseline. Moreover, the result showed that a transient increase in the number of CTCs prior to achieving a PR suggested transient tumor cell intravasation due to the nivolumab treatment and its potential correlation with the duration of the response, depending on its magnitude, compared to that at the baseline. These observations may promote setting the avenue for diagnosis with minimal invasiveness using CTCs and for precision medicine of the PD-1/PD-L1 blockade in lung cancer.

## Figures and Tables

**Table 1:** Patient characteristics.

Characteristic	Variable	Number
Number of Patients		*N* = 44
Age: median, (range) years		68 (49–86)
Gender: *n* (%)	Male	33 (75)
	Female	11 (25)
Smoking history: *n* (%)	Ever smoker	30 (68)
	Never smoker	14 (32)
Histological type: *n* (%)	Adenocarcinoma	30 (68)
	-EGFR mutated	9 (20)
	Squamous cell carcinoma	11 (25)
	Other	3 (7)
Stage: *n* (%)	III	11 (25)
	IV	33 (75)
Performance status: *n* (%)	0	9 (20)
	1	30 (68)
	≥2	5 (11)
Previous therapies: *n* (%)	0	1 (2)
	1	21 (48)
	2	13 (30)
	≥3	9 (20)

## Data Availability

The data presented in this study are available from the corresponding author upon reasonable request. The data are not publicly available due to maintain patient confidentiality.

## Supplementary Materials

The following are available online at https://www.mdpi.com/article/10.3390/cancers13102290/s1: Figure S1: Change in the CTC status between the baseline and progressive disease (PD) for patients defined as PD until week 4 or at week 4 for non-PD patients beyond week 4. Figure S2: Prediction of the long-term efficacy based on the PD-L1 positivity rates in the CTCs at weeks 4 and 12. Figure S3: Transition in total circulating tumor cells (CTC) counts in patients with immune-related adverse events (irAE). Table S1: Clinicopathological findings and CTC enumeration.
