# Supplementary material for: Longitudinal Evaluation of PD-L1 Expression on Circulating Tumor Cells in Non-Small Cell Lung Cancer Patients Treated with Nivolumab
Source: Cancers (Basel). 2021 May 11;13(10):2290. doi: 10.3390/cancers13102290 (PMC8150706; doi:10.3390/cancers13102290)
Supplement: Supplementary file 1 [file cancers-13-02290-s001.zip › cancers-1208026-supplementary.pdf]

Supplementary Materials: Longitudinal Evaluation of PD-L1 Expression on Circulating Tumor Cells in Non-Small Cell Lung Cancer Patients Treated with Nivolumab

Mio Ikeda, Yasuhiro Koh, Shunsuke Teraoka, Koichi Sato, Jun Oyanagi, Atsushi Hayata, Nahomi Tokudome, Hiroaki Akamatsu, Yuichi Ozawa, Katsuya Endo, Masayuki Higuchi, Masanori Nakanishi, Hiroki Ueda, Nobuyuki Yamamoto

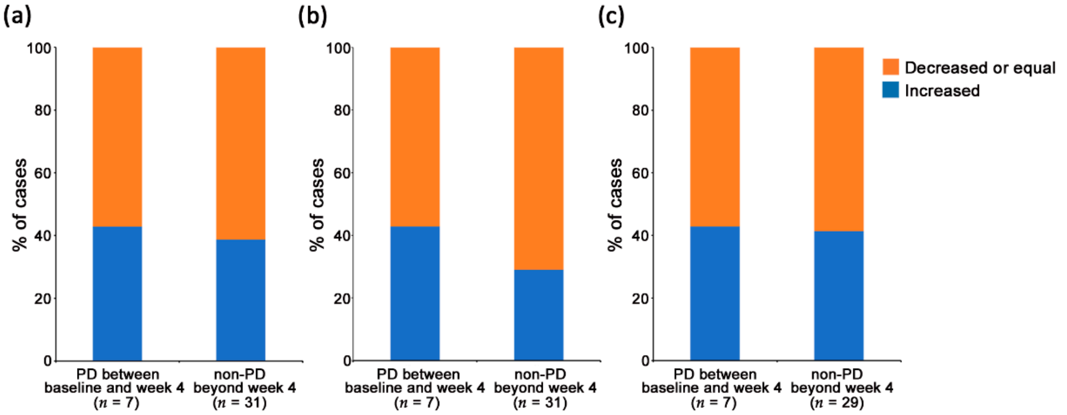

Figure S1. Comparison of changes in circulating tumor cells (CTCs) status between patients who developed progressive disease (PD) until week 4 (*n* = 7) and who continued to benefit from nivolumab treatment (non-PD) beyond week 4 (*n* = 31). The increased/decreased or equal of each CTC status in PD patients was compared between baseline and at point of disease progression and that in non-PD patients was compared between baseline and at week 4. The association of changes in (**a**) the total number of CTCs, (**b**) number of programmed death-ligand 1 (PD-L1)-positive CTCs, and (**c**) PD-L1 positivity rates upon nivolumab treatment were compared between PD and non-PD patients. Orange indicates that each CTC status at point of disease progression or week 4 was decrease or equal compared to that at baseline, while blue indicates increase.

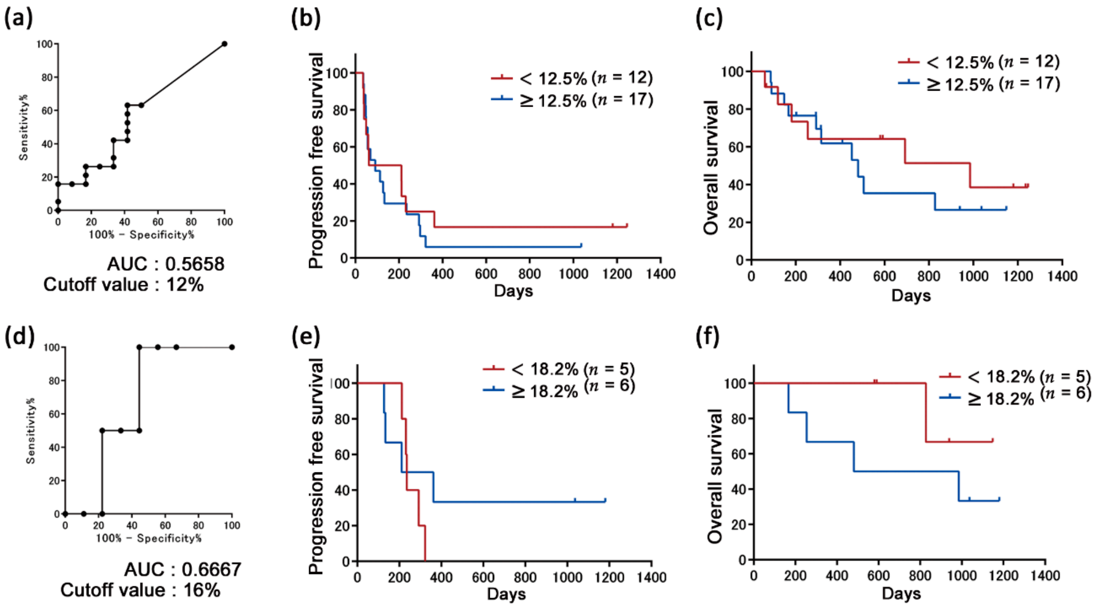

Figure S2. Prediction of long-term efficacy based on the ratio of programmed death-ligand 1 (PD-L1)-positive circulating tumor cells (CTCs) at week 4 and 12. (**a**) Cutoff value of PD-L1 positivity rates in CTCs at week 4 to segregate durable clinical benefit (DCB; *n* = 11) from non-DCB (*n* = 18) according

to receiver operating characteristic (ROC) curve. (**b**) Kaplan–Meier curve for progression-free survival (PFS). There was no significant difference in patients with ≥12.5% PD-L1 positivity rate (n = 17; median, 91 days) than in those with <12.5% PD-L1 positivity rate (n = 12; median, 136.5 days) at week 4. p = 0.53 (**c**) Kaplan–Meier curve for overall survival (OS) at week 4. No significant difference in OS between patients with ≥12.5% PD-L1 positivity rate (n = 17; median, 481 days) and in those with <12.5% PD-L1-positivity rate (n = 12; median, 985 days) at week 4. p = 0.50. (**d**) Cutoff value of PD-L1 positivity rates in CTCs at week 12 to segregate DCB (n = 9) from non-DCB (n = 2) according to ROC curve. (**e**) Kaplan–Meier curve for PFS. There was no significant difference in patients with ≥18.2% PD-L1 positivity-rates (n = 6; median, 268.5 days) than in those with <18.2% PD-L1 positivity rate (n = 5; median, 235 days) at week 12. p = 0.41 (**f**) Kaplan–Meier curve for OS at week 12. No significant difference in OS between patients with ≥18.2% PD-L1 positivity rates (n = 6; median, 733 days) and in those with <18.2% PD-L1 positivity rate (n = 5; median, not reached) at week 12. p = 0.25.

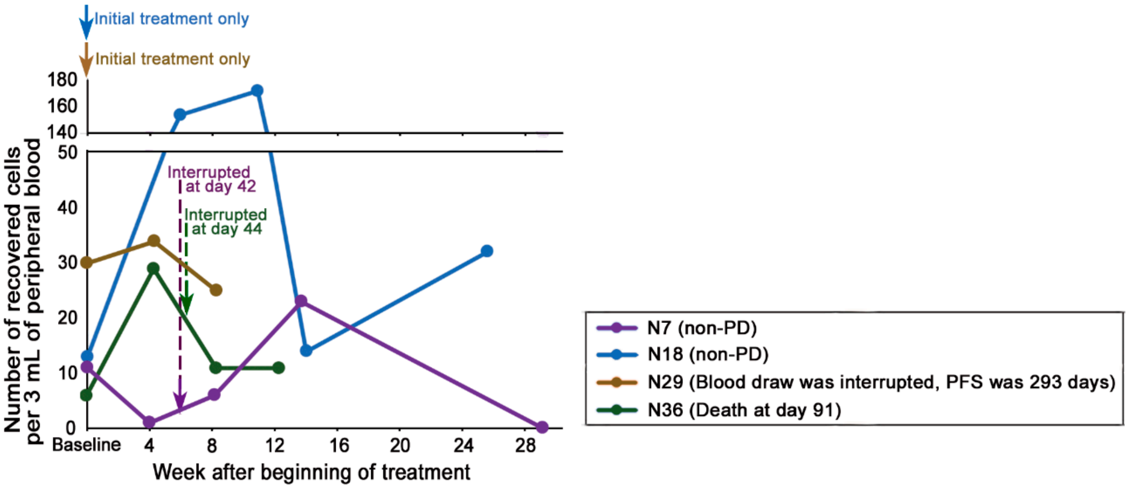

Figure S3. Transition in total circulating tumor cells (CTC) counts in patients with immune-related adverse events (irAE) who interrupted treatment in earlier phase. CTC counts in patients with irAE did not increase significantly after nivolumab interruption. Violet line indicates the total CTC counts in patient N7 who benefiting from nivolumab treatment until recent [non-progressive disease (PD)]. Nivolumab treatment was interrupted because of pneumonitis at day 42, and steroids were administered at day 55. The best response and progression-free survival (PFS) of this patient were partial response (PR) and 1246 days (aborted), respectively. CTC counts in this patient increased drastically prior to achieving PR. Blue line indicates the total CTC counts in patient N18 who also benefited from nivolumab treatment until recent (non-PD). Nivolumab was administered only as the initial treatment, interrupted for pneumonitis, and steroids were administered at day 14. The best response and PFS of this patient were PR and 685 days (aborted), respectively. CTC counts in this patient increased drastically prior to achieving PR. Brown line indicates the total CTC counts in patient N29. Nivolumab was administered only as the initial treatment, interrupted for pneumonitis, and steroids were administered at day 22. The best response, PFS, and overall survival (OS) of this patient were stable disease (SD), 217, and 293 days, respectively; however, blood draw was interrupted at week 8. Green line indicates the total CTC counts in patient N36. Nivolumab treatment was interrupted for drug-induced pneumonia at day 44, and steroids were administered at day 53. The best response, PFS, and OS of this patient were SD, 91, and 91 days, respectively.

Table S1. Clinicopathological findings and CTC enumeration.

Pt#	Gender	Age	Histological Type	Stage	Previous Therapies	Best Response	Number of CTCs / 3 mL blood						Remarks
							Baseline	Week 4	Week 8	Week 12	Week 24	PD	
							Total CTC / PD-L1-CTC	Total CTC / PD-L1-CTC	Total CTC / PD-L1-CTC	Total CTC / PD-L1-CTC	Total CTC / PD-L1-CTC	Total CTC / PD-L1-CTC	
N1	M	74	Adenocarcinoma	IIIB	3	PD	90	na	na	na	na	na	
			EGFR L858R				10 (11.1%)	na	na	na	na	na	
N2	M	79	Adenocarcinoma	IV	3	PD	23	na	na	na	na	82	
			EGFR exon19deletion				7 (30.4%)	na	na	na	na	0 (0.0%)	
N3	F	79	Adenocarcinoma	IV	2	PD	26	3	na	na	na	2	
			EGFR exon19deletion				6 (23.1%)	0 (0%)	na	na	na	0 (0.0%)	
N4	M	67	Adenocarcinoma	IV	2	SD	8	0	21	0	0	5	
							4 (50.0%)	0 (-%)	0 (0.0%)	0 (-%)	0 (-%)	2 (40.0%	
N5	M	50	Adenocarcinoma	IV	4	SD	5	12	12	na	na	25	
							4 (80.0%)	4 (3.33%)	1 (8.3%)	na	na	1 (4.0%)	
N6	M	53	Squamous cell carcinoma	IIIB	1	SD	19	4	3	12	na	7	
							12 (63%)	3 (75.0%)	0 (0.0%)	5 (41.7%)	na	1 (14.3%)	
N7	M	68	Squamous cell carcinoma	IV	2	PR	11	1	6	23	0	non-PD	Steroid administration after week4

							0 (0.0%)	0 (0.0%)	0 (0.0%)	17 (73.9%)	0 (-%)	
N8	M	80	Adenocarcinoma	IIIB	1	PR	15	1	16	11	na	3
							1 (6.7%)	1 (100.0%)	1 (6.3%)	2 (18.2%)	na	0 (0.0%)
N9	F	68	Adenocarcinoma EGFR L858R	IV	4	PD	42	na	na	na	na	24
							9 (21.4%)	na	na	na	na	0 (0.0%)
N10	M	73	Adenocarcinoma	IIIA	1	SD	16	2	10	na	na	2
							1 (6.3%)	0 (0.0%)	0 (0.0%)	na	na	2 (100.0%)
N11	M	76	Adenocarcinoma	IV	2	PR	na	na	na	na	na	na	All samples were not appropriate.
							na	na	na	na	na	na	
N12	F	49	Adenocarcinoma	IV	1	PD	13	4	na	na	na	2
							5 (38.5%)	0 (0.0%)	na	na	na	0 (0.0%)
N13	M	71	Adenocarcinoma	IV	0	NE	41	na	na	na	na	na
							14 34%)	na	na	na	na	na
N14	M	71	LCNEC	IV	5	PD	9	3	na	na	na	5
							0 (0.0%)	0 (0.0%)	na	na	na	2 (40.0%)
N15	M	68	Squamous cell carcinoma	IV	2	PD	22	26	na	na	na	11
							6 (27.3%)	16 (61.5%)	na	na	na	7 (63.6%)
N16	M	54	Adenocarcinoma	IIIB	2	CR	4	4	13	449	29	non-PD

							2 (50.0%)	0 (0.0%)	1 (7.7%)	393 (87.5%)	25 (86.2%)		
N17	M	76	Squamous cell carcinoma	IV	1	PD	13	4	na	na	na	1	
							6 (46.2%)	1 (25.0%)	na	na	na	0 (0.0%)	
N18	M	75	NOS	IV	2	PR	13	153	171	14	32	non-PD	Steroid administration after baseline
							8 (61.5%)	46 (30.1%)	30 (17.5)	8 (57.1%)	25 (78.1%)		
N19	M	83	Adenocarcinoma	IV	2	PD	7	12	na	na	na	10	
							2 (29%)	9 (75.0%)	na	na	na	4 (40.0%)	
N20	F	73	Adenocarcinoma	IV	1	SD	1	9	2	2	7	4	
							0 (0.0%)	4 (44.4%)	0 (0.0%)	0 (0.0%)	0 (0.0%)	3 (75.0%)	
N21	M	69	Unkown	IV	1	SD	27	36	9	34	3	na	
							12 (44.4%)	11 (30.6%)	1 (11.1%)	5 (14.7%)	0	na	
N22	M	62	Adenocarcinoma	IV	2	na	11	na	na	na	na	na	Transferred after baseline
							3 (27.3%)	na	na	na	na	na	
N23	M	86	Squamous cell carcinoma	IV	1	PR	38	10	2	0	17	na	
							25 (65.8%)	7 (70.0%)	1 (50%)	0 (-%)	13 (76.5%)	na	
N24	F	75	Adenocarcinoma	IV	4	PD	9	15	na	na	na	4	
			EGFR L858R				1 (11.1%)	2 (13.3%)	na	na	na	3 (75.0%)	

N25	F	64	Adenocarcinoma	IV	2	PR	34	3	18	na	na	na	Transferred after week 8
							11 (32.4%)	1 (33.3%)	0 (0.0%)	na	na	na	
N26	M	553	Squamous cell carcinoma	IIIA	1	SD	34	11	21	11	12	non-PD	
							22 (64.7%)	8 (72.7%)	7 (33.3%)	7 (63.6%)	11 (91.7%)		
N27	M	68	Adenocarcinoma	IV	1	NE	2	na	na	na	na	16	
							1 (50.0%)	na	na	na	na	10 (62.5%)	
N28	M	65	Adenocarcinoma	IV	1	PD	72	na	na	na	na	25	
							14 (19.4%)	na	na	na	na	8 (32.0%)	
N29	F	73	Squamous cell carcinoma	IV	1	PR	30	34	na	na	na	na	Steroid administration after baseline
							9 (30.0%)	31 (91.2%)	na	na	na	na	
N30	F	64	Adenocarcinoma	IIIB	3	PR	2	4	4	49	11	na	
							2 (100.0%)	0 (0.0%)	1 (25.0%)	11 (22.4%)	9 (81.8%)	na	
N31	F	77	Adenocarcinoma	IV	1	PD	12	24	na	na	na	9	
							1 (8.3%)	3 (12.5%)	na	na	na	4 (44.4%)	
N32	M	54	Adenocarcinoma	IIIB	2	PD	3	81	na	na	na	1	
							2 (66.7%)	56 (69.1%)	na	na	na	1 (100.0%)	

N33	M	64	Squamous cell carcinoma	IV	1	PD	3	3	na	na	na	2	
							3 (100.0%)	0 (0.0%)	na	na	na	0 (0.0%)	
N34	M	78	Adenocarcinoma	IV	1	PD	104	37	na	na	na	44	
							1 (1.0%)	6 (16.2%)	na	na	na	32 (72.7%)	
N35	M	57	Squamous cell carcinoma	IIIA	1	SD	28	115	139	32	8	na	
							14 (50.0%)	14 (12.2%)	84 (60.4%)	7 (21.9%)	3 (37.5%)	na	
N36	M	75	Adenocarcinoma	IV	3	SD	6	29	11	11	na	na	Steroid administration after week 4
			EGFR exon19deletion				5 (83.3%)	12 (41.4%)	6 (54.5%)	1 (9.1%)	na	na	
N 37	M	60	Squamous cell carcinoma	IIIB	2	SD	24	15	276	116	35	na	
							4 (16.7%)	8 (53.3%)	142 (51.4%)	8 (6.9%)	9 (25.7%)	na	
N38	F	68	Adenocarcinoma	III	1	PD	2	na	na	na	na	1	
							0 (0.0%)	na	na	na	na	1 (100.0%)	
N39	M	86	Adenocarcinoma	IV	2	PD	2	4	na	na	na	1	
			EGFR L861Q				1 (50.0%)	0 (0.0%)	na	na	na	0 (0.0%)	
N40	M	62	Adenocarcinoma	IV	5	NE	77	na	na	na	na	na	

			EGFR L858R				22 (28.6%)	na	na	na	na	na
N41	F	63	Adenocarci noma	IV	2	PD	5	na	na	na	na	83
							2 (40.0%)	na	na	na	na	15 (18.1%)
N42	M	83	Adenocarci noma	IV	1	SD	2	2	1	2	0	0
							0 (0%)	0 (0.0%)	0 (0.0%)	0 (0.0%)	0 (-%)	0 (-%)
N43	M	68	Adenocarci noma	IV	1	PR	27	40	3	2	1	1
			EGFR L858R				0 (0.0%)	0 (0.0%)	0 (0.0%)	0 (0.0%)	0 (0.0%)	0 (0.0%)
N44	M	65	Squamous cell carcinoma	IIIB	1	PD	50	na	na	na	na	3
							0 (0.0%)	na	na	na	na	0 (0.0%)
N45	M	49	Adenocarci noma	IV	1	SD	11	0	na	na	na	3
							0 (0.0%)	0 (-%)	na	na	na	2 (66.7%)
